# Bibliographical Notices

**Published:** 1851-11

**Authors:** 


### BIBLIOGRAPHICAL NOTICES.

*A Dictionary of Medical Science, containing a concise explanation of the various subjects and terms of Physiology, Pathology, Hygiene, Therapeutics, Pharmacology, Obstetrics, Medical Jurisprudence, &c., with the French and other Synonymes; Notices of Climate, and of celebrated Mineral Waters; Formulæ for various Officinal, Empirical and Dietetic preparations, &c.* By ROBLEY DUNGLISON, M. D., Professor of the Institutes of Medicine in Jefferson Medical College, Philadelphia. *Eighth Edition, revised and greatly enlarged.* Philadelphia, Blanchard & Lea, 1851.

In the present edition of this popular work, the author has included *four thousand terms* not found in the last, and *has* made it, "not only a satisfactory and desirable," but "indispensable lexicon," in which the student may find every term now legitimated in the nomenclature of Medical Science. It is the most enduring monument to his industry and research.

---

*Surgical Anatomy.* By JOSEPH MACLISE, Surgeon. With sixty-eight colored Plates. Philadelphia, Blanchard & Lea, 1851.

Part V. and last of this admirable reprint, has just been issued by Messrs. Blanchard & Lea, and is now offered to the professional public.

In notices of the previous parts, we have called attention to the excellence of this work, and have recommended it to the student of medicine, and practitioner, removed from the schools, as presenting a truthful series of dissections demonstrative of the relative anatomy of the principal region of the human body. From this candid expression, the present part does not incline us to swerve; it fully equals its predecessors in beauty and faithfulness of execution. It contains the conclusion of the demonstration of the urinary organs, and comprises in plates 63 and 64, deformities of the urinary bladder, the operations of sounding for stone, of catheterism, and of puncturing the bladder above the pubes.

In the commentary on this plate, there will be found one of the clearest descriptions of the mode of introducing the catheter, that we ever seen. Plates 65 and 66 illustrate the surgical dissection of the popliteal space and the posterior crural region. Plates 67 and 68, the surgical dissection of the anterior crural region, the ankles, and the foot. In bringing to a close our notices of the various parts of Mr. Maclise's work, we desire to express our conviction of its excellence, as regards both plates and commentary. That it is without faults, we do not pretend to say; but they are so few in comparison with its merits, that they can be readily forgiven. To the publishers also, our acknowledgments are justly due, for the liberal expenditure they have made upon it, as well as for the exceedingly low rate at which it is offered. With all these good qualities, we are confident it will find a place in every surgeon's library.

---

### *The Physicians' Visiting List, Diary, and Book of Engagements for* 1852.

Messrs. Lindsay & Blakiston have just published a most convenient little book with the above title, containing blanks for twenty-five daily visits, memoranda, &c., for the use of physicians. With it in his pocket, the practitioner has a list of his engagements, and his day book, and diary. We commend it to the profession from practical experience, as the most complete thing of the kind we have ever seen.

*A system of Operative Surgery, based upon the Practice of Surgeons in the United States, and comprising a Bibliographical Index and Historical Record of many of their operations during a period of two hundred years.* By Henry H. Smith, M. D., Surgeon to the St. Joseph's Hospital, &c. &c. *Illustrated by numerous Steel Plates.* Philadelphia: Lippincott Grambo & Co., 1851.

We have merely had time to glance over this recent publication, the mechanical execution of which reflects great credit upon all connected with it. The illustrations are on steel, and beautifully colored. Of the text, we have not as yet had time to judge. In our next number we purpose to lay before our readers a critical digest of its contents.

---

*Elements of Physiology, including Physiological Anatomy.* By Wm. B. Carpenter, M. D., F. R. S., F. G. S., &c., &c. *Second American, from a new and revised London Edition. With one hundred and ninety Illustrations,* Philadelphia: Blanchard & Lea, 1851.

The Elements of Physiology of Dr. Carpenter is one of a series of manuals, published in England, and intended for the use of students more especially. Although it embraces, in a great degree, the scope of the other treatises by the same author, it cannot be regarded as a mere abridgment of them, being, so far as it goes, complete in itself. The present edition has been greatly improved, much of it having been re-written, particularly the chapters on the vital force, and those on generation, and the nervous system. Several new cuts are found in it, and the student will find it in every particular fully brought up to the existing state of the science. We commend it to him as one of the best elementary treatises on the subject.

